# Obstetrics, Gynecology, and Abdominal Surgery

**Published:** 1900-09

**Authors:** William Warren Potter

**Affiliations:** Buffalo, N. Y.


### OBSTETRICS, GYNECOLOGY, AND ABDOMINAL SURGERY.

CONDUCTED BY WILLIAM WARREN POTTER, M. D., Buffalo, N. Y.

### TREATMENT OF RUPTURE OF THE PARTURIENT UTERUS.

WENDEL (*Medical Record*, May 26, 1900,) says: There is no dividing line that can be determined between threatened and beginning rupture. Is it justifiable, then, to use forceps in high degrees of thinning? Koblank found that 10 of 80 cases of rupture were caused by forceps. Winckel and Schaeffer found that high forceps give worse results to mother. Two distinct rules are applicable to head presentations when rupture threatens.

1. When thinning of the lower segment is slight and the round ligaments are tense only during contractions, deliver with forceps.

2. When Bandl's ring is very high or very oblique, and one or both of the round ligaments are tense between pains, delivery should be effected by Cesarean section or craniotomy.

The same rule holds good for version in head presentations, the hand being substituted for forceps. The rule of action in transverse presentations is simpler, for when the child is living this fact indicates that dangerous thinning has not yet occurred and version may be attempted with deep narcosis. When rupture has occurred, delivery must be accomplished without increasing the injury. In head presentation with living fetus in utero, prompt application of forceps may save the child without increasing the mother's dangers, provided the os is completely dilated and no pelvic contraction exists. When the head remains in the uterus and the body has escaped, McLane argues that the danger of increasing the injury no longer exists, and recommends passing the hand along the child until a limb can be grasped, then rotate and deliver, instead of losing time by attempting to grasp the mobile head with forceps. A dead fetus would, of course, be delivered by perforation and cranioclasty. Spontaneous rupture with transverse presentation results almost invariably in fetal death. Embryotomy would therefore take precedence when the fetus remained either entirely or in greater part in the uterus. If rupture occurs during version the operator must judge whether he had better continue or not; in general the child should have a chance for life. When the child escapes entirely into the peritoneal cavity, the conjugate diameter measuring less than 8 cm., abdominal section will be necessary.

### PUERPERAL INFECTION.

H. W. LONGYEAR (*Canada Medical Record*, July, 1900,) states that the early diagnosis and treatment of pseudo-membrane by topical applications is of great importance.

The intrauterine douche frequently applied is of the most value in the forms of infection unattended by the formation of a pseudo-membrane. The vaginal use of peroxide of hydrogen is helpful in all forms of infection. Frequent packing of the vagina previously dried with iodoform gauze is especially useful in cases with pseudo-membrane. In the general treatment he gives quinine twice daily, whiskey and strychnine to support the heart, nuclein and protonuclein in all cases, mercurial and saline cathartics at first in all cases, then as indicated. Serum therapy to be applied where the Klebs-Löffler bacillus or the streptococcus can be demonstrated by the bacteriologic examination. Streptococcus antitoxin serum to be used persistently to prevent pus formation and symptoms of systemic infection.

---

### PREVENTION OF CONCEPTION.

IN A discussion of this delicate theme before the Hamburg Obstetrical Society (*Centralblatt für Gynakologie*, 1900, No. 18) various methods were proposed, none of which appeared to be certain. Objections were made to so-called occlusive pessaries, since they frequently give rise to endometritis, and were harmful in cases of inflammatory disease of the pelvic organs. It was agreed that under some conditions the physician was justified in trying to prevent conception.

In cases of extreme pelvic contraction, tuberculosis and other wasting diseases, it was advised that ligation or resection of both tubes must be practised when the abdomen was opened for other reasons.—*Am. Jour. of Med. Sciences.*

---

### DYING DECLARATIONS IN ABORTION CASES.

THE Supreme Court of New Jersey (*Jour. of the American Medical Association*) holds, in the case of State vs. Meyer, that, upon an indictment framed under the provisions of section 119 of the Crimes Act of 1898, which charges the defendant with using an instrument upon the person of a pregnant woman, with intent to cause her miscarriage, and that the defendant's act caused the death of the woman, evidence of the dying declarations of the deceased is inadmissible. The reason given is that death is, in New Jersey, no longer in any case an essential element of the crime denounced, but is to be considered solely with respect to the punishment to be inflicted after conviction, which the court holds renders inadmissible the dying declarations.

---

INDIVIDUAL SIGNS OF PREGNANCY.

O. NAEGELE (*Medical and Surgical Monitor*, August, 190c,) has observed that women who have previously suffered from phlebitis or thrombosis of the veins of the lower limbs, especially in the saphenæ, have a prompt and reliable pregnancy barometer in their varix. Some multipara can affirm their pregnancy in eight days after conception from the condition of their varix. Women who have had puerperal complications with exudates and formation of adhesions, frequently experience a revival of symptoms at these points immediately after conception, the locus minoris resistentiæ proving an index of pregnancy. In two cases a thrush formation was noted on the external genitals, causing pain on urinating. He concludes his article in the *Muench. Med. Woch.*, of June 12th, calling attention to the valvular formation at the external orifice of the urethra in women, which forms a right-angled closure with a slit in the middle. It is most pronounced in virgins and in persons who have never masturbated.

---

INJURY TO THE SKULL IN NARROW PELVES.

HERMANN (*Allg. med. Central-Zeitung*, February 17, 1900,) exhibited at the recent meeting of the Schlesische Gesellschaft für vaterländische Cultur a child with a marked funnelshaped impression upon the vertex acquired in connection with the operation of turning and extracting.

The woman was an VIII-para. No history of rickets. Five of the eight labors required operative interference, either turning or perforation. The ninth labor set in very deliberately. On account of failure of child to advance, the author's help was invoked. Child in second vertex position. Somewhat pendulous abdomen. Head movable in superior strait. Diagonal conjugate measured 11 cm.

A hypodermic of morphia was given for the exhaustion, to enable the mother to regain some of her lost strength. The head soon afterward engaged in typical occipital presentation. Here was an indication for version. On account of early rupture of water this operation was difficult, and energetic traction was necessary before the child could be extracted. Child born asphyxiated, but was revived. The want of proportion between fetal skull and maternal pelvis had left its mark —a deep funnel-shaped impression over the right parietal measuring 6½ cm. long and 3½ cm. broad. The pelvis was found to be of the simple flat variety, with a true conjugate of 9.5 cm.

The child is now 10 weeks old, and has had no trouble of any sort. No external or internal hemorrhage had occurred.

Comparing the present state of the child's head with photographs made eight days after labor, we see that the lesions has considerably improved, but there can be no doubt that it is destined to be permanent through life.

An inspection of the literature of the subject shows that these impressions are of very infrequent occurrence. Ahlfeld has seen but ten cases in 3,000 labors. The prognosis is better than in other forms of injury to the cranial bones—fissures, fractures, etc. Remote effects involving the development of the encephalon are unknown.

This lesion is typical of the result of extracting the aftercoming head in contracted pelvis. A short, violent effort of traction is requisite for its production. Such an experience can be eminently dangerous for a child, and the favorable issue of the author's case should not make us oblivious of the usual prognosis in such instances (mortality in version in contracted pelves 36 per cent.)—*Obstetrics*, July, 1900.

---

PREGNANCY IN THE FIMBRIATED EXTREMITY OF THE FALLOPIAN TUBE.

DR. W. B. DORSETT (*St. Louis Courier of Medicine*, August, 1900,) presented to the Obstetrical and Gynecological Society a specimen of a fetus from this condition. The patient had been curetted by another physician for a supposed miscarriage; following this the abdomen became very much distended and tense. When seen by him, the patient had a temperature of 102°F., and a very weak pulse while a mass filled the lower abdomen as high as the umbilicus. Pelvic abscess was diagnosticated and the patient was prepared for operation. Under anesthesia, a large retrocele was found occupying the vagina, which gave a feeling of fluctuation. When incised, a large quantity of clotted blood escaped. Introducing the hand, a fetus was removed, together with a quart and a half of clotted blood. The ovary could not be outlined, but a ragged portion of the tube could be felt near the fimbriated extremity and it was assumed that this was a case of gestation in the fimbriæ. Nothing was found having the appearance of placental tissue. Patient's temperature and pulse immediately improved and she recovered, though a purulent discharge continued for some weeks. In this case there was no history of sterility, often found in cases of extrauterine pregnancy, no history of shock, or of a rupture, or of fainting; no sudden pain, though there were pains simulating those of uterine contractions which misled the patient and her physician into the belief that she had miscarried and for which he had curetted the uterus, removing what was undoubtedly the decidua.

